# Comparison of Intralesional Meglumine Antimonite along with oral Itraconazole to Intralesional Meglumine Antimonite in the treatment of Cutaneous Leishmaniasis

**DOI:** 10.12669/pjms.35.6.363

**Published:** 2019

**Authors:** Uzma Bashir, Moizza Tahir, Muhammad Irfan Anwar, Faisal Manzoor

**Affiliations:** 1 Dr. Uzma Bashir, MCPS, FCPS. Department of Dermatology and Otolaryngology, Combined Military Hospital, Quetta, Pakistan; 2 Dr. Moizza Tahir, MCPS, FCPS, MHPE. Department of Dermatology, Combined Military Hospital, Multan, Pakistan; 3 Dr. Muhammad Irfan Anwar, FCPS. Department of Dermatology, PNS Shifa, Karachi, Pakistan; 4 Dr. Faisal Manzoor, MCPS, FCPS. Department of Dermatology and Otolaryngology, Combined Military Hospital, Quetta, Pakistan

**Keywords:** Cutaneous leishmaniasis, Itraconazole, Intralesional meglumine antimoniate, IL = Intralesional,, MA = Meglumine Antimoniate,, LD = Leishmania Donovan,, CL = Cutaneous leishmaniasis,, CMH = Combined Military Hospital

## Abstract

**Background & Objective:**

Cutaneous leishmaniasis (CL) is endemic in developing countries like Pakistan. Pentavalent antimonials are still drug of choice, despite being toxic and intolerable for patients. Second line treatments have been extensively studied but the results of their efficacy are conflicting. This, to our knowledge, will be the first study in this regard. Our objective was to determine if combination of oral itraconazole with intralesional (IL) meglumine antimoniate (MA) reduces the duration of treatment for cutaneous leishmaniasis, as compared to intralesional MA alone.

**Methods:**

A randomized controlled trial (single blinded) was carried out from August 2017 till December 2017 on 69 patients who fulfilled inclusion criteria. They were assigned to Group-A or B by lottery method. Group-A patients received IL MA once a week while Group-B received oral itraconazole 200mg, once daily, for six weeks along with similar regimen of IL MA as Group-A. The patients were assessed every three weeks by the blinded assessor till clinical cure was achieved. A follow up visit, two months after clinical cure was done to look for relapse of the disease.

**Results:**

Thirty patients in Group-A and 35 patients in Group-B completed the study. At 3, 6, 9 and 12 weeks the patients were assessed for: no, partial or complete response and results of the two groups were compared for statistical significance. The p-values of 0.20, 0.57 and 0.11 at 3, 6 and 9 weeks, respectively, depict that there was no significant difference at any step of assessment between the two groups in terms of healing. The p values of each t test was>0.05 refuting the hypothesis.

**Conclusion:**

Combination of oral itraconazole with intralesional MA offered no benefit over intralesional MA alone in the management of cutaneous leishmaniasis in terms of duration of therapy.

## INTRODUCTION

Cutaneous Leishmaniasis (CL) is a neglected protozoal disease with a worldwide incidence of around 1.5 million cases per year according to World Health Organization.^1^ It is currently a serious public health problem in developing countries like Pakistan where the incidence is on the rise due to immigrants across the country,^2^ military deployments, humanitarian aid workers and expanding infrastructure in the endemic areas.^2^,^3^ It is caused by more than 20 species of Leishmania, the vector in old world being Phlebotomus sand fly. Humans are accidental hosts and can act as reservoir of infection. It is recommended that all patients infected with CL should be treated to reduce the prolonged course of disease, to reduce scar formation and to eliminate the reservoir of infection.^4^ Management of CL is still being done with pentavalent antimonials despite being toxic and intolerable for most patients. The search for safer oral alternatives that would lead to an early cure of the disease and elimination of amastigotes in human reservoirs has led to a range of second line treatments including azole antifungals, miltefosine, dapsone, imiquimod, azithromycin etc.

The role of itraconazole has been specifically proven of benefit, as a second line treatment, in CL in old world.^4^ For lesions that do not qualify systemic therapy with pentavalent antimonials, intralesional injection of meglumine antimoniate is given with or without other topical or destructive procedures.^5^ There still exists a literature gap on trials combining second line treatments with intralesional MA. In our quest for an effective early cure of the disease, we examined the combined effect of oral itraconazole with intralesional meglumine antimoniate, to see if the duration of therapy could be reduced or the combined effect produced better cosmetic result. This combination has not been tried before in an experimental study and our study will guide future practice in this regard. Our objective was to see if oral Itraconazole along with intralesional meglumine antimonite will shorten the duration of treatment for CL as compared to intralesional MA alone.

## METHODS

Patients presenting to CMH Quetta were all soldiers serving in various parts of Balochistan and belts extending north till Waziristan where L. tropica is more prevalent as compared to L. major.^3^ Inclusion criteria were: patients between 16-60 years of age, patients with positive smear or skin biopsies for amastigotes, lesions four or less in number and no lesion more than 4cm in size. Pregnant or lactating women, sporotrichoid spread, use of any anti-leishmania treatment in the past 2-3 months, lesions at sites that merit systemic antimonials, allergy to antimonials and patients with history of liver disease were excluded from the study.

### Ethical Approval

The study protocol and consent form were approved by the Ethical Review Board of the hospital. (Approval December 12, 2018)

It was a Randomized single blind control trial, conducted from July 2017 till December 2017 in CMH Quetta, a tertiary care hospital. Patients with skin lesions suggestive of leishmaniasis had slit smears taken for Leishmania Donovan bodies, those with negative smears underwent skin biopsy for parasitological confirmation. This process was completed in five days. They were then enrolled in the study after taking informed consent.

At enrollment, a demographic profile of each patient was made and they underwent a complete examination of the skin lesions by the assessor. The duration of lesions at enrollment, number of CL lesions, their size and location were charted. A baseline photograph of the skin lesions was also taken. Assessor was blinded in group-allocation and treatment regimen of patients. However, if the patient’s disease progressed at any step, the dermatologist assigned on assessment would shift the patient to systemic antimonial therapy.

Randomization was done by lottery method to divide the patients into Group-A or B. A box with 70 slips containing equal number of the two groups was offered to the patients. Those in Group-A received intralesional Glucantime (Tillots pharma, Switzerland), 1ml/cm^2^ of the lesion, weekly till the lesions healed. Group-B received capsule Itraconazole (Cap Icon Ferozsons Pakistan) 200mg daily for six weeks along with the same intralesional Glucantime regimen as Group-A. Intralesional Glucantime was given by another dermatologist to all the patients, to remove the bias of injection technique. An independent physician (a third doctor) was given the task to provide cap icon to Group-B patients on fortnightly basis for six weeks, he also monitored patients for side-effects, ordered serum LFTs once in the middle of treatment and also checked their compliance by asking how they were taking the medicine. Duration of treatment was defined as the number of days to complete healing from the day of enrollment. The study end point was defined as complete clinical cure of the skin lesions or lost to follow up or shift to systemic therapy for an individual patient. Clinical cure was defined as complete absence of inflammation (erythema/induration/crusting) and re-epithelialization of the ulcer.

The status of each lesion was reassessed by the blinded assessor every three weeks till she declared the lesion healed (till complete clinical cure). At each visit the patients were categorized as having;

1. No response
2. Partial response
3. Complete response

The decision to shift a patient, if any, to systemic antimonials also lied with the assessor. The patients were reviewed for follow up by the assessor two months after treatment ended to rule out relapse, defined by recurrence of induration/erythema in the scar.

**Table I:** Group comparison on treatment response at three weeks’ assessments. 1= No response, 2=Partial response, 3= Complete response.

	Treatment group	N	Mean	Std. Deviation	Std. Error Mean
Response at 3 wks.	A	30	1.07	0.254	0.046
	B	35	1.17	0.382	0.065
Response at 6 wks.	A	28	1.93	0.262	0.050
	B	35	1.89	0.323	0.055
Response at 9 wks.	A	30	2.07	0.254	0.046
	B	34	2.21	0.410	0.070
Response at 12 wks.	A	28	3.00	0.000^a^	0.000
	B	34	3.00	0.000^a^	0.000

a. t cannot be computed because the standard deviations of both groups are 0.

### Statistical analysis

The results were analyzed by SPSS version 23.

## RESULTS

Between 1^st^ August till 30^th^ December, 2017, 114 patients were screened positive for cutaneous leishmaniasis. Of these, 69 (95% confidence level and 7.4% confidence interval) patients who filled our inclusion criteria were included in the study. However, within the first two weeks three patients were shifted to systemic antimonial treatment due to development of newer lesions or sporotrichoid spread and one patient withdrew from the study due to his deployment in another city. Among the 65 patients, 30(46.2%) completed the trial in Group-A and 35 patients (53.8%) in Group-B. 64 (98.5%) were males and one (1.5%) was female. All the males (64) were soldiers deployed in various regions of Balochistan while the female was a resident of Quetta. There was no statistically significant difference in demographic characteristics and lesion features (location and mean size of lesions) between the two groups at the start of treatment. The lesions on lower limb were most common (47.7%) in both the groups while those on trunk were least (7.7%). The mean duration of lesions in Group-A was 4.46 weeks (SD ± 1.16) while in Group-B it was 4.65 weeks (SD ± 1.10). The mean size of lesions in Group-A was 3.87cm ± 1.2 SD, while in Group-B it was 3.71 cm ± 1.02 SD.

On assessment of treatment response for both the groups at 3, 6 and 9 weeks the p-values of 0.20, 0.57 and 0.11 respectively, depict that there was no significance difference at any step of assessment between the two groups in terms of healing. The lesions showed complete clinical cure on 12^th^ week assessment in both groups, it reached 100% by once a week regimen, hence no ‘t scores’ could be obtained for that assessment. All the patients completed the duration of treatment till cure in both the groups and came for follow up around two months after being declared cured. No patient in either group had to stop treatment because of any side effects. Minimal to no derangement in S. LFTs was observed in Group-B and their treatment was not withheld. There was no difference in the characteristics of scar formation, as observed by the assessor, between the two groups. There was no recurrence of lesions on 18^th^ week follow up in both the groups, hence there was no edge of one treatment over another in this regard.

**Table II:** Independent sample t-test scores for both groups at 3-weekly assessment.

		Levene’s Test for Equality of Variances		t-test for Equality of Means				

		F	Sig.	t	df	Sig. (2-tailed)	95% Confidence Interval of the Difference	

							Lower	Upper
Response at 3 wks.	Equal variances assumed	7.308	0.009	-1.278	63	0.206	-0.269	0.059
	Equal variances not assumed			-1.317	59.494	0.193	-0.264	0.054
Response at 6 wks.	Equal variances assumed	1.331	0.253	0.568	61	0.572	-0.108	0.194
	Equal variances not assumed			0.581	60.978	0.563	-0.105	0.190
Response at 9 wks.	Equal variances assumed	12.190	0.001	-1.606	62	0.113	-0.313	0.034
	Equal variances not assumed			-1.652	55.852	0.104	-0.308	0.030

**Fig. 1:**
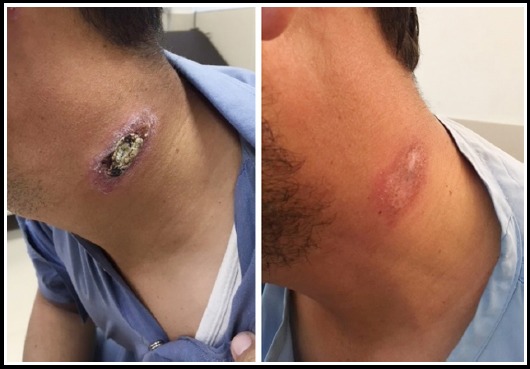
Group-A

**Fig. 2:**
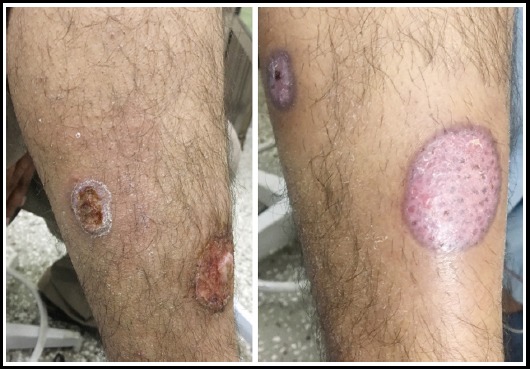
Group-B

## DISCUSSION

Cutaneous leishmaniasis of old world is endemic in Pakistan. The areas especially affected are Balochistan, Khyber Pakhtunkhwa and Waziristan, however, incidences occur in interior Sindh, Kashmir and urban cities like Multan.^3^,^6^-^8^ Parenteral antimonials, mostly meglumine antimoniate, remain the cornerstone of treatment. The MA injections are toxic and intolerant for most patients due to pain at injection site, fever and arthralgias. There is a need for more effective, less time consuming and tolerable treatment modality for this disease.^9^ Alternatives have been extensively studied and reported but the results are conflicting for some modes of treatment.^10^,^11^ The studies on efficacy of intralesional MA as compared to IM injections of MA, established that both are equally effective in achieving cure^10^ while MA is more effective (82%) than Sodium stibogluconate (67%) when given IL.^12^ Cryotherapy, carbon dioxide laser, thermotherapy, paromomycin cream, zinc sulphate as intralesional injections have been used with variable success rates.^9^,^13^-^15^ The role of oral azole therapy, specifically itraconazole, in systemic treatment is also very well established.^16^-^18^ Despite being effective, there is not enough evidence to support the exclusive use of azoles as a single treatment mode in leishmaniasis.^19^

The outcome of interest in these studies, like in our study, was an early clinical cure, defined as complete re-epithelialization of all lesions. In Balochistan leishmaniasis season is late September through October when bulk of patients report. Of the 114 patients who reported to our hospital, 69 fulfilled the criteria for topical treatment as per the WHO guidelines.^5^ Keeping the extensive data on efficacy of itraconazole use in clearing CL lesions, we hypothesized that combined therapy of intralesional MA with oral itraconazole would reduce the duration of therapy for intralesional MA towards clinical cure. The cure rate with IL MA had been estimated at about 80% after one month of twice a week regimen (8 injections).^12^ In our study, partial cure was achieved in 94.2% patients by 9^th^ injection. In another study the duration to cure for once a week regimen of IL MA was 6.2±0.7 weeks,^13^ which is in contrast to our study that showed a cure at 10±1.2 weeks. The duration of treatment till cure with itraconazole 200mg for six weeks was 75% in another study,^4^ while in a meta-analysis the efficacy rate was 65% for itraconazole in old world CL.^19^ Since we did not examine the efficacy of itraconazole alone but the response rate at six weeks in Group-B was partial (100% patients) in our study. Another study comparing the cure rate of combination of itraconazole with dapsone, imiquimod or cryotherapy to monotherapy with these agents showed that monotherapy gave an overall success rate of 56.41%, whereas combination therapy was successful in 69.56% of patients.^18^ We achieved an equal success rate in both groups on the contrary because our monotherapy included IL MA.

The time to healing in each patient was noted separately by the assessor every three weeks. The p-value of test scores in both groups at each three weekly visit was > 0.05, which refuted our hypothesis that the combination treatment would reduce the duration of treatment. Since the patients were all soldiers except one, their follow up visit two months after cure could easily be arranged. None of the patients showed recurrence after two months, which proved that there was no advantage of the combination therapy in this aspect too. Another variable we, unintentionally, noted in our study was the rate of conversion to systemic antimonials while on IL MA treatment, which was 3/69 patients (4.3%).

### Limitation of study

Leishmania specie identification was not done in this study which can alter the treatment response. The study is not adequately powered.

## CONCLUSION

Combination of oral itraconazole along with intralesional injection of MA offered no benefit over intralesional Glucantime alone in the management of cutaneous leishmaniasis.

### Authors` Contribution:

**UB** conceived, designed, did statistical analysis and editing of the manuscript.

**MT** did data collection, record of lesions and editing of manuscript.

**MIA** did statistical analysis and review of literature.

**FM** did data collection and review of literature and article.
